# Advantages and Disadvantages

**Published:** 1917-11-03

**Authors:** 


### Advantages and Disadvantages.

Lieut.-Colonel D'Arcy Power, (1st London General Hospital) said he had seen a good deal of the method and the way in which it was used, and he was able to confirm, from the surgeon's point of view, the complete satisfaction it had given. It seemed to him, however, that in other than skilled and careful hands the degree of anæsthesia was liable to be on the light side, and so to cause trouble.

Dr. Dudley Buxton congratulated Captain Boyle on his paper for a number of reasons. In his view the use of nitrous oxide and oxygen in major surgery had been neglected in this country; comparatively few men had obtained that experience of the method which would enable them to obtain the best results from it. He emphasised the importance of the use being only in the hands of the expert, as he knew of no form of anæsthetic which required greater personal knowledge and skill in the administrator, especially as there was a liability towards too light a degree of anæsthesia. In America he considered the method had been used too indiscriminately, without observing the limitations which Captain Boyle detailed. He did not agree there should be cyanosis during narcotisation; when it was present there were worse after-effects. The amount of re-breathing needed by any particular patient taxed the judgment of the administrator to the full. The necessity for absolute silence prior to and during anæsthesia he could not emphasise too much.

Mr. H. J. Paterson spoke very highly of the method as carried out by Captain Boyle in his cases at the Queen Alexandra Hospital for Officers at Highgate, and exhibited, by means of the epidiascope, charts of cases showing the absence of shock or elevated temperature immediately following operations. One difficulty, which he hoped would soon be overcome, was that after abdominal operations a good deal of pain was experienced, possibly because the gas and oxygen did not fully paralyse the intestines, and their movements recommenced quickly. It might be a blessing in disguise by obviating that dread complication in these cases—ileus. It was in London that gas and oxygen was first used, and he was surprised when attending the American Congress in 1907 to hear the President of the Surgical Section claim to have discovered the method.

Col. H. J. Waring pointed out how great an economiser of the surgeon's time the method proved to be. It was very suitable in all short septic cases, and for all patients who were septic and very ill. After major operations, such as amputations, there was little shock, and it was very suitable for old people and those subject to bronchitis, for whom, on that account, the administration of ether or chloroform was risky. It was unsuitable for muscle-splitting operations, especially those in the upper part of the abdomen, as for the exploration of the gall bladder. For appendix operations, however, it was quite straightforward, and was very suitable for removal of enlarged prostates. Subsequent recovery after this form of anæsthesia was certainly excellent.

Dr. Chaldecote thought there could be no doubt that the after-effects when gas and oxygen was the form of anæsthesia were very much less disagreeable than by any other method. Crile's teaching that the local anæsthetic produced the anæsthesia, the gas and oxygen causing the unconsciousness. He thought the anæsthetic should not be withdrawn immediately the operation was at an end; and he deprecated moving the patient from one room to another after he had been narcotised. With this method much of the surgeon's trepidation on approaching such cases as those for excision of the rectum had been removed.

Major McAdam Eccles, M.S., also spoke favourably of the method, and suggested some small disadvantages, such as the weight of the apparatus (60 lb.). For the pain following abdominal sections he found hot water in a rubber bottle placed on the abdomen formed a more potent relief than the giving of morphia. He thought there should be a means of warming the gases before they were inhaled. He fully agreed as to the need for quiet during the induction of anæsthesia, and he would forbid the patient being touched at that time; certainly no bandage or splint should then be removed. He narrated some of his own cases.

Dr. Shipway thought that as C.E. had been used in association with the method the name given was a misnomer. Mr. Page, in his paper on the subject, admitted he used ether in 66 per cent. of his cases. He thought the re-breathing aspect needed to be carefully worked out in a scientific way, especially as $CO_2$ was a modifier of anæsthesia, and stimulated the respiratory centre. This was probably at the bottom of the tendency to vomit. Experiments on dogs showed that symptoms of cardiac failure followed an attempt to "crowd on" the nitrous oxide.

Mr. Bellamy Gardner said one great danger in attempting to rely entirely on gas and oxygen was the possibility of the occurrence of sickness at any moment, which, in an already partially asphyxiated patient, might put the final touch to the asphyxia by causing closure of the larynx. It became a duty to point out that this was the great danger in the hands of the inexperienced. He thought many of the deaths which had occurred, and would occur, were connected with this actual and impending sickness during the administration.

Dr. Hugh Phillips did not think that, on the whole, the method was of particular use in military surgery, especially in abdominal cases. Much of its effect depended on the local anæsthetic with which it was combined. Silence during induction was probably more important with this means than with any other anæsthetic.

The President (Sir St. Clair Thomson) referred to the advantage of the Society being able to bring together for such a debate both surgeons and anæsthetists, the result of which must tend to the advancement of knowledge. He spoke of his own experiences, and said how indebted he felt to anæsthetists in the dangerous and difficult operations which he had had to carry out.

